# Sustainability assurance practices: a systematic review and future research agenda

**DOI:** 10.1007/s11356-021-17359-9

**Published:** 2021-11-17

**Authors:** Saddam A. Hazaea, Jinyu Zhu, Saleh F. A. Khatib, Ayman Hassan Bazhair, Ahmed A. Elamer

**Affiliations:** 1 School of Accounting, Yunnan University of Finance and Economics, Kunming, 650221 China; 2 Azman Hashim International Business School, Universiti Teknologi Malaysia, 81310 Johor Bahru, Malaysia; 3 Faculty of Business Administration College, Department of Economic and Finance, Taif University, Taif, Saudi Arabia; 4 Brunel Business School, Brunel University London, Kingston Lane, Uxbridge, UB8 3PH London UK; 5 Department of Accounting, Faculty of Commerce, Mansoura University, Mansoura, Egypt

**Keywords:** Sustainability assurance, Internal audit, External audit, Audit committee, Environmental assurance, Sustainable development, Scopus

## Abstract

Although firms increasingly publish sustainability reports, assuring such reports is relatively new. This study reviews the literature of sustainability assurance to evaluate the intellectual development of the field and provide recommendations for future studies. It also demonstrates the role of assurance to enhance the credibility of sustainability reports and corporate reputation. This paper systematically reviews 94 papers obtained from the Scopus database between 1993 and August 2021. Our study shows that there is an increase in the number of studies published in recent years. We also found that some countries have received limited attention, such as the USA. The scant literature examining sustainability assurance in private institutions and non-profit organisations should be reinforced. Likewise, the sustainability research also provides limited evidence on the governance debate. The vast majority of research is not based on theoretical grounds. The need for assurance of sustainability reports not only enhances the reputation but also adds more value to the organisation’s planning, monitoring, and accountability. We highlight several new research suggestions that may enhance the understanding of sustainability assurance practices.

## Introduction

Sustainability is a topic of growing importance worldwide (Alshbili et al. 2021; Liu et al. 2021; Orazalin & Mahmood 2019). The effort of the UN Intergovernmental Panel on Climate Change (IPCC) on global warming and environmental laws and regulations has evolved rapidly in the last few years. The pace of change continues to increase to pursue a sustainable model of capitalism. Pressures from the community for better corporate behaviour has also increased due to highly publicised corporate environmental disasters (Kamran et al. 2021; Liu et al. 2021; Roberts et al. 2021a, 2021b; Shad et al. 2020). This trend has increased the demand for more environmental information and activities. Likewise, concerns related to the environmental and social effects of business have led to increased demand and desire to apply transparency on the entirety of issues related to corporate behaviour (Kamran et al. 2021; Khan et al. 2021). Some researchers confirmed that organisations participating in sustainability activities and disclosure would enhance transparency, reputation, and branding, encouraging employees and increasing competitiveness (Agyemang et al. 2020; Alshbili et al. 2021; Elmagrhi et al. 2019; Hassan et al. 2020; X. Chen et al. 2020; Song et al. 2021). Yet, a nascent, but growing stream of research has criticised the transparency and suitability of these practices.

The importance of sustainability engagement on its three dimensions is widely presented in the literature (social, environmental, and economic). Environmentally, increased human and industrial impact on the surrounding ecosystems has resulted in environmental changes, which have developed into one of the most significant issues in this era. Hence, sustainability has emerged as a critical factor in achieving environmental balance (Yadav et al. 2021). The importance of sustainability is demonstrated via efforts to maintain the environment and conserve natural resources, all of which contribute to a better quality of life. All companies try to embrace sustainability because of its importance in preserving resources and creating value in use (James 2014). Burhan and Rahmanti (2012) claimed that sustainability benefits investment returns and financial performance by producing value and thus ensuring the stability of earnings. Socially and economically, sustainability practices lead to a reduction in spending by providing savings, which leads to the use of these savings in supporting economic activities and local addition to investments. Moreover, communities that have good development plans are more attractive to investment and investors. Besides, good sustainability strategies reduce costs associated with the personal aspects of the consumer’s health. In addition, sound strategies for sustainability contribute to increasing productivity by employees and the surrounding environment becomes more encouraging for production and research aspects.

A key component of developing and implementing a sustainability plan in a corporation would require a periodic review of that plan and its implementation. An extensive body of research has demonstrated the role of audit that reviews sustainability from the side of environmental management and performance, and the relation of the environment to other ethical, labour, and social aspects. The importance of assurance in emphasising sustainability has been commonly highlighted as the most important mean of mitigating the risk of environmental violations and ensuring sustainability engagement of corporations (Coyne 2006; Desimone et al. 2020). According to the Institute of Internal Auditors (IIA 2021), audit functions add value to enterprises by strengthening risk management and improving the understanding of new emergent issues such as sustainability (Abdelfattah et al. 2020; El-Dyasty & Elamer 2020; Owusu et al. 2020). Additionally, Chiang and Torng (2015) argued that the audit component is the key point in the complete infrastructure under which the organisation’s interaction with changes in the environment can be managed. Ridley et al. (2011) argued that there is a growing recognition of the importance and necessity for organisations to report on issues related to sustainability, but the importance and value of such reports, which are acted upon impartially and independently, appear to be underappreciated by stakeholders. Hence, organisations have a rapid increase in sustainability assurance activities due to the need to monitor sustainability risk and activities (Fraser et al. 2020). Sustainability assurance includes three main characteristics: assessing the environment by using measurable standards and linking them to performance, relying on an audit team with sufficient accounting and financial experience and issuing reports of both types, internal to shareholders and external to the public (Nitkin & Brooks 1998). Sustainability assurance measures the value of organisations from three aspects: economic, social, and environmental (Coyne 2006). Trotman and Trotman (2015) reported that assurance is associated with sustainability in achieving accountability towards stakeholders.

A substantial number of prior research has documented that the interest of organisations in all countries has increased in sustainable development, which required an increase in the need for sustainability auditors and the application of assurance standards (Coyne 2006; Handoko et al. 2020; Silvola & Vinnari 2021). While there are some specific mandatory sustainability reporting instruments across the world, there are few regulations around sustainability reporting and none regarding sustainability assurance practices (Desimone et al. 2020). The assurance providers’ professionalism, independence, and quality of assurance statements have all been called into question in terms of their content analysis of sustainability reports and assurance statements. Furthermore, as sustainability assurance is costly, assurance statements mostly consist of theoretical reflections rather than field investigations. Sustainability assurance has, therefore, received growing attention from academics in recent years. Despite this trend, to date, relatively few review studies exist discussing the role of assurance in achieving and assuring sustainability practices (Csutora & Harangozo 2017; Qingliang Tang 2019). Our study, therefore, aims mainly to evaluate the prior studies that discussed assurance and sustainability topics to answer three main research questions.

- **RQ.1**. How was the research related to sustainability assurance developed and investigated?
- **RQ.2**. What is the current evaluation of previous studies (focus and criticisms)?
- **RQ.3**. How do future research on the role of assurance in sustainability can be identified?

In order to address these questions, a systematic literature review (SLR) method was followed to collect relevant studies from the Scopus database in the sustainability assurance area. Following Khatib et al. (2021b), a search string was developed with limited keywords identified after reviewing studies that discussed assurance or accounting with sustainability (i.e. Ridley et al. 2011). A final sample of 94 studies was included in the current study, which explicitly discusses assurance and sustainability.

A nascent, but growing research stream has focused on assurance and sustainability in recent times due to the desire of institutions to use assurance functions in assessing sustainability risks. However, a dearth of research on certain developed and developing countries was found. For instance, only one study in the USA has been found in the sample literature despite the large impact of assurance and sustainability on its economy. This indicates that there is a need for more research investigating sustainability assurance in both developed and developing countries. The review also reveals that the need for assurance of sustainability report does not only enhance the reputation of institutions but also can add more value to the organisation’s planning, monitoring, structure, and accountability. Furthermore, a significant number of practical works relied severely on archival data, pointing to the need for more reliable and valid hand-collected research, although some constructs that influence sustainability assurance cannot be easily observed using archival databases (e.g. psychology, culture, and preference of auditor). This study provides several other recommendations for future research directions.

Our paper contributes to this field as the first study, to our knowledge, that relied on SLR to provide a comprehensive and up-to-date review of the sustainability assurance literature. This research is useful for many stakeholders such as management, auditors, regulators, and researchers as it provides insights into the intellectual development of the sustainability assurance fields. Also, it shows the great importance of assurance and its contribution in enhancing the practice of sustainability to the extent of the stakeholders’ desire and confirms that there is a major role for assurance in emphasising sustainability as the most important means contributing to mitigate the risk related to environmental violations. Lastly, based on our SLR and the synthesis of research, this study outlines several avenues to be addressed in investigating sustainability assurance in future. In doing so, we build possible future research queries for the interactions of internal and external assurance, audit committees, and other types of assurance. The resulting future research avenues tie the study of assurance and sustainability to avoid perpetuating the divide and parallel examination endeavours.

The rest of this article is prepared as follows. The “Methodology” section summarises the methodology applied in this research including journal selection and content analysis. In the “Results and discussion” section, we present and discuss the results of the sample literature evaluation. The “Future research avenues” section highlights several suggestions for future work. We end with a conclusion in the “Conclusions and recommendations” section.

## Methodology

The systematic literature review approach is popular in management, finance, and economic fields (Hedin et al. 2019). Systematic literature review (SLR) can provide significantly unbiased results compared to traditional narrative review (Hazaea et al. 2021b; Kotb et al. 2020). Subjective and biased results can be reduced, and the investigation status is improved in the topic being discussed using SLRs (Massaro et al. 2015) as it limits scholars’ preference during the identification of the sample literature. Studies based on the SLR can confirm the transparency of the analysis with the possibility of replication (Easterby-Smith et al. 2015), and it differs from traditional reviews in that it follows strict and explicit rules in the way it is prepared (Massaro et al. 2016).

According to Kotb et al. (2020), Zhao et al. (2021), and Massaro et al. (2016), studies based on SLRs required the following steps: determine the protocol to be used for the review and identify the databases from which the research sample can be obtained (e.g. Scopus, Web of Science, Google Scholar, and ProQuest), determine the research questions to be answered using previous studies under investigation, determine the type of studies to be investigated and specify the time period, measure the impact of the article based on predefined rules (e.g. use the Google Scholar or Scopus citation to determine the high article impact among the readers), define the analytical framework for the studies, and use the developed framework to critically analyse prior literature to show the intellectual development of the field and highlight gaps by analysing previous steps to provide some avenues for future research. This approach was also used in several recent SLR studies (i.e. Hazaea et al. 2021a, b; Zamil et al. 2021; Zhao et al. 2021).

**Fig. 1:**
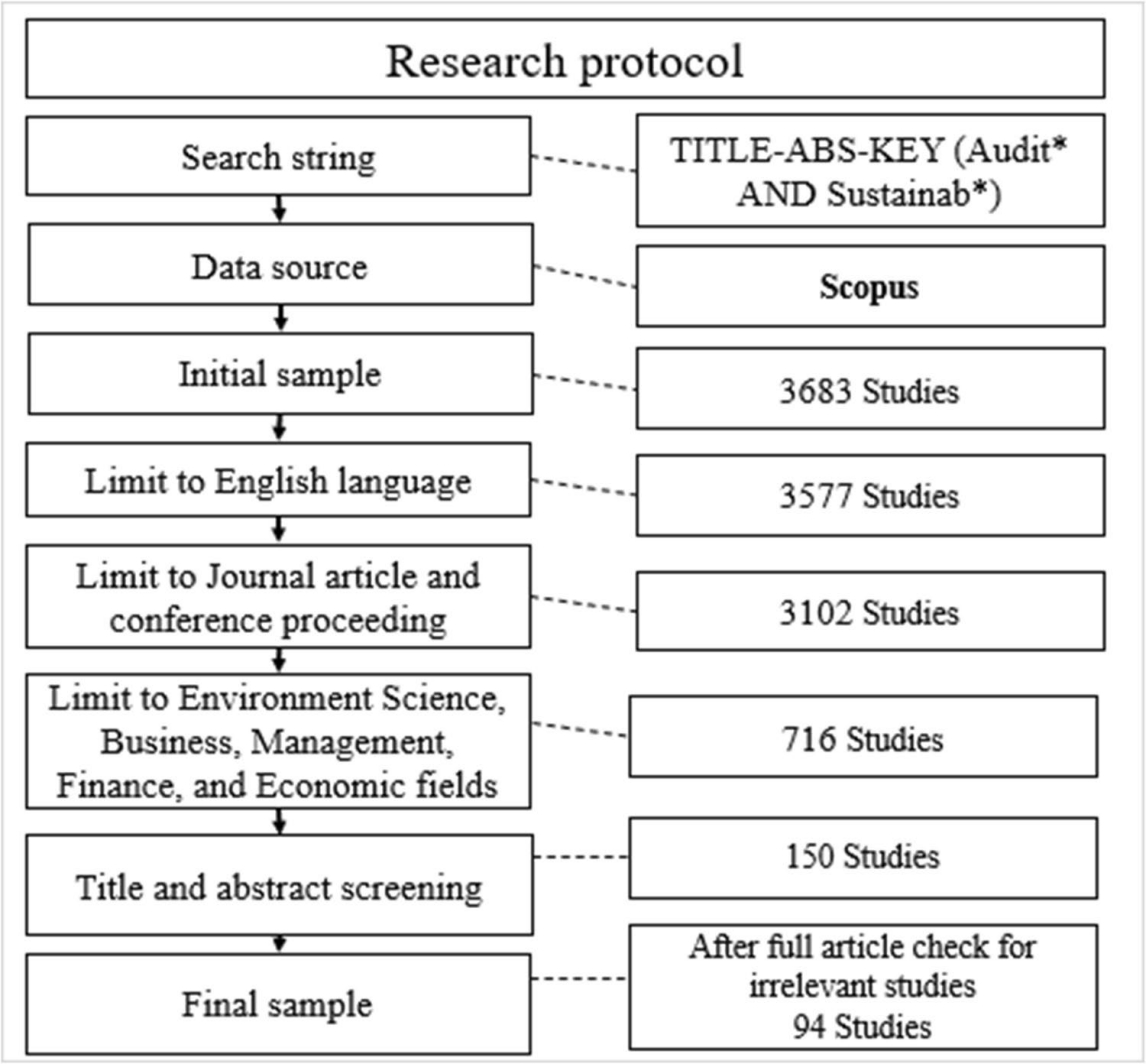
The flow chart of the sample collection

To avoid errors in implementing SLR, we followed some studies that discussed topics similar to the current study (e.g. Ascani et al. 2021; Khatib et al. 2021b; Nerantzidis et al. 2020; Widmann et al. 2021). The data collection was conducted in August 2021. We relied on the Scopus database to obtain the sample literature as it is the largest indexed abstract database compared to the others (Nerantzidis et al. 2020; Yahaya et al. 2020). As shown in Fig. 1, two keywords were utilised to search for studies related to the topic under study, namely “Audit*” and “Sustainab*”. It should be noted that the use of asterisk helped us to look for other similar terms such as auditor, auditing, audits, sustainability, sustainable. These keywords were used to search in the title, keywords, and abstract of the literature. The initial sample of studies hit the number of 3683 studies. Then, this sample was limited to studies that were published in the English language (result in 3577 studies) and in journal or conference proceedings (result in 3102 studies). Since all research included in the sample literature are carefully evaluated, publications in non-English languages were excluded due to our lack of language skills. Given that assurance and sustainability are the main focus of the study, we limited the search to studies that were published under the **“**Environment Science” **OR** “Business” **OR** “Management” **OR** “Finance” **OR** “Economic**”** subjects which significantly reduce the sample literature to 716 studies. Finally, we screened the titles and abstracts of the final number of documents, and articles that are irrelevant to assurance and sustainability were excluded, resulting in 150 articles. After a thorough analysis of all publications that directly addressed sustainability assurance, a final sample of 94 research was included in this study.

### Research questions

Our questions are based on three basic criteria: (i) knowing the current status of the studies under investigation, description, and objective, (ii) direct criticism of current studies, evaluating and identifying gaps, and (iii) guide future research to cover the gaps. According to extant research (i.e. Kotb et al. 2020; Nerantzidis et al. 2020), for studies based on SLR, three questions should be included. In this study, the first question provides an investigation of how the studies that discussed assurance and sustainability have evolved. According to Dumay and Garanina (2013) and Tsalavoutas et al. (2020), this question can be addressed through several points such as assessment of the most influential paper, regional distribution, quality of journals, the affiliation of authors, research setting, and research instrument. The second question can be answered by analysing the main research topics, the theoretical basis, and evaluating the previous literature results (see, Khatib et al. 2021a; Kotb et al. 2020; Massaro et al. 2016). The third question is addressed by highlighting the avenues for future work during the process of addressing the first and second questions.

## Results and discussion

### Descriptive analysis

#### Yearly trends

**Fig. 2:**
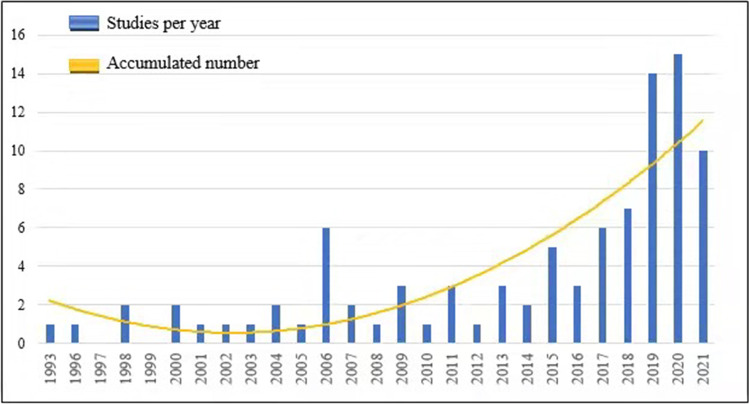
The yearly trend of the published documents

In Fig. 2, it seems clear that the number of studies that discussed assurance and sustainability during the period 2015 to 2021 has increased significantly, especially in 2020, where the number of published studies was 15 articles. The increase in studies that discussed assurance and sustainability in the recent period may be due to the desire of institutions to use audit functions in assessing sustainability risks. Besides, the financial crises added momentum to the demand for stricter rules and regulations, more transparent disclosure, and greater management accountability. Fraser et al. (2020) emphasised that there is an increase in the trend of sustainability assurance by institutions and organisations due to the need to use assurance functions in monitoring sustainability risks and their activities. Similarly, Ahmed (2016a, b) emphasised that as a result of the increase in social responsibility in all aspects, including the economy, the interest of auditors in the social responsibility of companies increased. Therefore, the increased orientation by the auditors led to the necessity of conducting research related to this activity.

In general, the interest of stakeholders has increased in the implementation of sustainability activities in addition to the interest of regulators and their encouragement for institutions in developing sustainable activities because of their importance to various economic aspects (Mensah 2019). For instance, Ridley et al. (2011) argued that sustainability assurances contribute to governance, risk management, and control of organisations in emerging markets. Consequently, we argue that increasing stakeholder interest in sustainability activities leads to increased consideration of the importance of assurance in improving sustainability activities, which requires further research to better understand the complex nature of the relationship between sustainability and assurance aspects, especially post COVID-19 crisis.

#### Regional distribution

In this section, we evaluated the geographical distribution of the sample literature. The investigation showed that the studies were distributed among 24 countries only, while 15 studies were cross country, and 23 were non-regional studies (Table 1). Due to the limited studies that belong to every country, we followed Ascani et al. (2021) and utilised the continental classification. Table 2 shows that China is the most investigated country in our sample with five articles [5.32%]. This could be attributed to the recent sustainable development policies taking place in China (Ascani et al. 2021). For example, a study by Tang (2019) showed that the most important factors that increased the practice of carbon assurance in China are the rapid expansion and development of carbon enterprises and the government’s encouragement of these enterprises. Canada came second with 4 studies, 4.26%, such as Nitkin and Brooks (1998), which discussed the experiences of Canadian companies in public and private sectors in integrating sustainable development management with preparing reports as an essential part of their business. The study indicated that the majority of companies operating in Canada do not review their environmental sustainability as the reports and practice of sustainable development auditing are not mandatory. In addition, the study results indicated that the practice of sustainability audit is based on many factors, the most important of which are legal responsibility before the state, corporate commitment, transparency associated with the audit process, and general awareness of environmental issues.

**Table 1:** Regional distribution of the sample studies

Country	Number	Continent	%
China	5	Asia	5.32%
Canada	4	NA	4.26%
Malaysia	3	Asia	3.19%
Romania	3	Europe	3.19%
The UK	3	Europe	3.19%
Taiwan	3	Asia	3.19%
Australia	3	Oceania	3.19%
Indonesia	3	Asia	3.19%
Spain	3	Europe	3.19%
Thailand	2	Asia	2.13%
India	2	Asia	2.13%
Ukraine	2	Europe	2.13%%
Vietnam	2	Asia	2.13%%
Finland	1	Europe	1.06%
Russia	1	Europe	1.06%
Saudi	1	Asia	1.06%
German	1	Europe	1.06%
Netherlands	1	Europe	1.06%
Zimbabwe	1	Africa	1.06%
Nigeria	1	Africa	1.06%
USA	1	America	1.06%
Iraq	1	Asia	1.06%
Fiji	1	Oceania	1.06%
Brazil	1	SA	1.06%
The EU	6	Europe	6.38%
GCC	1	Asia	1.06%
Cross country	15	-	15.96%
No country	23	-	24.47%

*NA*, North America; *SA*, South America; *GCC*, Gulf Cooperation Council; *EU*, Europe

**Table 2:** The top 10 leading articles in the sample literature

Author/s	Citation	Country/method	Research focus
Simnett et al. (2009)	567	31 countries 2113 firms ML-SLA	Determining the factors related to the voluntary purchase decision, selection and confirmation of the assurance provider, and revealing the importance of the assurance to support the need for companies to enhance credibility and selection of the assurance provider
Rennings et al. (2006)	315	German 1277 survey-interviews Descriptive	Discuss the impact of characteristics of assurance scheme and environmental management on economic performance and the innovations of technical environmental
Casterella et al. (2004)	169	USA 651 questionnaires ML and OLS	Analysing competition strategies that can create a sustainable environment in Big-6 audit firms and the impact of non-specialist auditors on that
Manetti and Becatti (2009)	154	Cross-county Reports Descriptive	Discussing assurance services in sustainability reports, with a focus on discussing the basic international standards that determine the implementation of assurance services, in addition to analysing the different types of assurance statements
Maijoor and Witteloostuijn (1996)	121	Netherlands Practical- reports Descriptive	The relationship of strategic organisations to audit and its impact on sustainability
Sierra and Zorio (2012)	86	Spain Reports Descriptive and Logistic regression	The auditor’s relationship in ensuring social responsibility
Gao and Zhang (2006)	80	Non-empirical Review	Investigating the extent to which social auditing can be applied in performance and sustainability assessment
Zeng et al. (2007)	75	China 156 questionnaires Descriptive	Effective auditing associated with the ISO 9001 standard and its role in achieving sustainability and creating competition
Morimoto et al. (2004)	70	Mixed method -review and interview	Investigating the possibility of developing a CSR audit system based on the viewpoint of stakeholders, practitioners and regulators
Wallage (2000)	66	Non-empirical review	The role of financial auditors in verifying sustainability reports
Ferreira et al. (2006)	55	Case study	The role of environmental auditing and management in promoting sustainability
Lebaron et al. (2017)	39	Cross country (China-USA and UK) Mixed method Descriptive	The role of audit in corporate accountability and sustainability

*SLA*, sequential logit analysis; *ML*, multiple regression; *OLS*, ordinary least squares

The results highlighted that some countries were investigated only three times in prior research, namely Malaysia, Romania, The UK, Taiwan, Australia, Indonesia, and Spain, while the rest of the countries were subject to less than two studies. Moreover, some researchers were interested in evaluating the role of economic and cultural differences by conducting cross-country research (15 studies). For example, a study by Guidara et al. (2021) aimed to assess the relationship between the effectiveness of assurance standards and sustainability by using a sample from 125 countries. Al-Matari and Mgammal (2019) reported that multi-country studies can provide basic and important insights that enable researchers to understand the subject under investigation from various cultural, social, and political aspects.

Furthermore, we grouped the sample literature into several categories based on the content distribution of the studies. We found that Asia ranked first in the number of studies with 23 articles (e.g. Charumathi & Krishnan 2011; Chiang & Torng 2015; Tang 2019), followed by Europe with 21 articles (Serbănică et al. 2015; Watson and Emery 2003), then South and North America with six articles (i.e. Oliveira et al. 2011; Rubenstein 2001), and Oceania with four articles, and Africa with 2 articles. Interestingly, there is only one study from the USA despite its large socio-economic impact. This indicates that there is a need for more research investigating sustainability assurance in both developed and developing countries that received less attention in the literature.

#### Leading research

Table 2 presents the most influential research studies that have an impact among sustainability assurance scholars based on the citation matrix provided by the Scopus database. The result revealed that the study conducted by Simnett et al. (2009) is the most influential research with 567 citations. The study determined the factors related to the voluntary purchase decision, selection, and confirmation of the assurance provider. It also revealed the importance of assurance in supporting the need for companies to enhance credibility and selection of the assurance provider. The study used a sample of 2113 companies in 31 countries, and the results showed that companies that aim to enhance their credibility with regard to stakeholders and aim to develop and build their corporate reputation often get confirmed reports of their sustainability. In addition, the study results indicated that the assurance provider might not be definitively linked to the audit profession. Likewise, a study by Rennings et al. (2006) also has another influential research with 315 citations. This study investigated the interrelationship between economic performance, technical environmental innovations, and environmental management and assurance scheme. It found that environmental process innovations are positively affected by the environmental management systems (Rennings et al. 2006). The reason may be that this paper was published long ago and it has been suggested that old studies may get more chances to be cited (Ascani et al. 2021; Kotb et al. 2020). The study conducted by Casterella et al. (2004) ranked third with 169 citations. This study analysed competition strategies that can create a sustainable environment in Big-6 audit firms and the impact of non-specialist auditors on that. The reason behind its impact among researchers may be because it was conducted in the world’s largest economies, in addition to the fact that this study was published a long time ago. In this context, we believe that studies published in high-impact journals and implemented in a strong economic region may have a significant impact among researchers as a result of the prevailing belief that research results are strong.

#### Distribution of the sample publication upon journals

Table 3 describes the sample of the studies under investigation based on the sources of journals in which the sample studies were published. We focused on studies that discussed assurance and sustainability and obtained 94 studies that were analysed. Following Chen et al. (2018) and Hristov et al. (2021), journals were divided into three categories. The first category is the journals categorised in the environment and sustainability; the second category is the journals categorised in accounting, operations management, and performance; and the third category is the journals categorised in general management. Table 3 shows that the second type included the largest number of 39 research articles, where this type was classified into five categories. *Managerial Auditing Journal* ranked first with 6 research papers, followed by *International Journal of Auditing: A Journal of Practice and Theory* and *Accounting Auditing and Accountability Journal*, 2 research papers for each journal. This confirms the extent to which audit functions are related to sustainability, especially that the research published in the first three journals, the majority of them after 2010 which indicates the interest of researchers in this field. In the second place came research published in the journals of environment and sustainability, where *Business Strategy and the Environment* has published six research papers, which ranks first in publishing research related to sustainability and topics related to management such as accounting. This indicates that this journal is a pioneer in publishing research related to management and the environment. Followed by the *Journal of Cleaner Production* with five research papers, then the *Journal of Business Ethics* that includes five research papers, and sustainability 4 research papers. These journals emphasise the important role that assurance plays in ensuring sustainability. Remarkably, most of the journals indicated in the table are among the best journals in assurance and sustainability, which confirm the importance of this research. Another interesting thing that confirms the growing interest in sustainability assurance is the presence of research published in the post-2017 period in journals with high impact among readers and pioneers in the disciplines of assurance and sustainability.

**Table 3:** Distribution of the articles sample by journals

Articles divided by journals	Number. of articles	%	Period of publications	Web of Science index	ABS* 2021
**First category**; sustainability and environment	**38**	**40.43%**	1993–2021		
*Business Strategy and the Environment*	6	6.38%	2006–2021	SSCI	3*
*Journal of Cleaner Production*	5	5.32%	1993–2021	SCIE	2*
*Journal of Business Ethics*	5	5.32%	1998–2019	SSCI	3*
*Sustainability*	4	4.26%	2017–2021	SCIE-SSCI	-
Other	18	19.15%	2001–2021		
**Second category;** Accounting, auditing, performance and operations management	**39**	**41.49%**	2000–2021		
*Managerial Auditing Journal*	6	6.38%	2000–2015	SSCI	2*
*Auditing; A Journal of Practice and Theory*	2	2.13%	2000–2004	SSCI	3*
*International Journal of Auditing*	2	2.13%	2018–2019	SSCI	3*
*Accounting, Auditing and Accountability Journal*	2	2.13%	2020–2021	SSCI	3*
Others	27	28.72%	1996–2021		
**Third category;** general management	**17**	**18.08%**	1998–2021		

### The analytical framework

#### Theories applied in the literature

Through the general analysis of the 94 studies included in the investigation, the investigation shows that there are only 44 studies that relied on theories and were distributed as follows: agency theory (3 articles), stakeholder theory (2 articles), legitimacy theory (2 articles), institutional theory (2 articles), other single theory (11 articles), and mixed theory (24 articles). Table 4 (A) shows the theories that were used in the studies under investigation, either individually or with other theories. Agency theory was used in 15 studies (see, Annuar & Abdul Rashid 2015; Wang et al. 2020). The analysis also shows that stakeholder theory has been used in 13 studies either individually or with other theories. Wang (2017) applied stakeholder theory to investigate the association between the characteristics of firms including the characteristics of the audit committee (AC) and disclosure of sustainability reports. The result showed that there is a positive association between characteristics of AC and disclosure of sustainability reports. Legitimacy theory was used in eight studies (e.g. Fernandez-Feijoo et al. 2018). The results showed that ensuring sustainability can be achieved to a large degree and a high level when the financial auditors belong to the Big4 audit firms. The institutional theory was used in 4 studies (e.g. Silvola & Vinnari 2021) where this theory was used as a basis for verifying the role of the agency and the management style in achieving sustainability assurance and the role of auditors in achieving this. The results showed that the refusal of the institutional work of other agents by the auditors might help in ensuring the achievement of sustainability.

**Table 4:** Analytical framework of the literature

A. Theories	# Studies	C. Organisation’s focus	# Studies
Stakeholder theory	13	Public sector	18
Agency theory	15	Publicly listed firms	13
Legitimacy theory	8	Private sector	2
Institutional theory	4	Mixed	8
Resource dependency theory	4	General/other and non-applicable*	53
Signaling theory	2		
Other theories*	20		
**B. Research methods**		**D. Sustainability definitions**	
Questionnaire/other empirical	16	Environmental-social-economic	52
Reports	32	Environmental-economic	23
Interview/case study	7	Environmental-social	16
Review/non-empirical-content analysis	32	Environmental	3
Mixed method	7		
**E. Assurance type (thematic)**			
Internal audit			7
External assurance			1
Audit committee			13
Environmental assurance			10
Another specialist assurance*			14
General audit			49

^*^Other theories including *stewardship, relational, chaos, transition management, fraud diamond, planned behavioural, the media setting agenda, social political theory, capability, social cognitive, industrial organisation, cost theory, organisational ecology, general system theory, ethical and corporate cultural theory, activity system theory, critical political economy theory, and carbon audit theory, grounded theory, and sustainability theory*. **General/other and non-applicable:** including *Big4, Big6 and IIA member, *etc.*Other specialist assurance: *carbon assurance, social assurance, environmental assurance, government audit, financial audit, and firm audit*

Resource dependency theory was also used in four studies. It should be noted that 24 studies used mixed theories (i.e. Rika 2009) where the aim was to investigate the incentives that call for the use of environmental assurance in the Fiji public institutions. The results showed that external and internal factors necessitated the use of environmental assurance, including the request of international organisations and the United Nations, in addition to the new laws in the country. The study results indicated that this could be explained by using the institutional and legitimacy theories.

It should be noted that the theories have been directed from three aspects: economic (critical political economy theory), social (ethical and corporate cultural theory and social-political theory), and psychological (theory of planned behavioural) and this reinforces the necessity of building research based on theories, which contributes to the development of research work. Thus, expanding the adoption of the theoretical basis in assurance research by using new theories based on economic, social, and psychological aspects enhances the audit functions towards sustainability assurance.

Interestingly, 50 research articles did not apply a theoretical framework in evaluating the topic (Kaziliünas 2008; Oliveira et al. 2011; Serbănică et al. 2015). It is worth noting that it might be difficult to understand the outcomes of any study that is not based on a theoretical basis (Nerantzidis et al. 2020). According to Beck and Stolterman (2016), studies which are not based on theories may be insufficient in providing insight into the topic in question. This is one of the limitations of previous studies under investigation and we, therefore, encourage future studies to consider this issue.

##### Stakeholder theory

The theory of stakeholders is one of the most important theories used as a major research approach related to sustainability management (Wang 2017). Numerous studies have established that stakeholder theory is a critical component in explaining sustainability and preparing financial reports (Belal & Roberts 2010; Reynolds & Yuthas 2008). The stakeholder theory assumes that corporations should take into account all the different expectations surrounding their business. Additionally, it emphasised the need for the management to identify the nature of the environment surrounding the performance of their institutions, including the regulation of the association between internal and external stakeholders. According to Hermawan and Gunardi (2019), stakeholders may affect the performance of companies through the impact of social ownership, profitability, financial leverage, and the independence of those authorised in the management of these institutions from the disclosure of social responsibility. This is consistent with what is supported by the stakeholder theory. In the sustainability assurance literature, this theory is used in different studies with a different framework such as the risk of CSR and auditors (Brooks et al. 2019), carbon auditing (Qingliang Tang 2019), sustainability development and corporate governance (Suttipun & Saelee 2015), and corporate sustainability and social assurance (Gao & Zhang 2004). The significance of stakeholder theory is that it guides stakeholders in general to commercial firms’ proper thinking. However, it does not incorporate the ethical concepts essential for managers to deal with some issues, such as those relating to the natural environment that do not clearly and directly involve individuals within commercial institutions (Orts & Strudler 2002). Despite the extensive application of this theory in research, there is still a dearth of literature on the subject.

##### Agency theory

It was applied in 15 studies. The agency theory is related to the conflict of interest resulting from the separation of ownership (Fama & Jensen 1983; Hazaea et al. 2020; Hazaea et al. 2021a; Khatib & Nour,). Agency theory is concerned with investigating problems that arise for one party in terms of decision-making and implementation of activities (Al Amosh and Khatib 2021; Eisenhardt 1989). The literature under investigation used agency theory to explore many areas such as auditor gender and crash risk (Wang et al. 2020), sustainable development and the type of gender of the member of the audit committee (Bravo and Reguera-Alvarado 2019), the impact of corporate governance on sustainability (Cancela et al. 2020; Suttipun and Saelee 2015), and the impact of audit committees (ACs) on corporate sustainability (Buallay and Al-Ajmi 2020). Although this theory is frequently used in the sample research articles, the interpretations are deemed inadequate. Especially with new and modern topics, given that the basics of this theory are old (Bendickson et al. 2016), some studies consider that the agency theory ignored many human motives while focusing only on the aspect of self-interest and human behaviour (Alshbili et al. 2019; Chariri 2008; Elamer et al. 2019, 2021).

##### Legitimacy theory

According to Rika (2009) and Zamil et al. (2021), the theory of legitimacy is one of the most used theories in research related to environmental accounting. However, there are many problems to apply and follow this theory, such as the necessity of economic work under conditions of competition, continuous and great pressure from stakeholders, in addition to fragmented social values (Neu et al. 1998). Tilling (2004) argued that the legitimacy theory could contribute significantly to providing a strong and systematic mechanism for the non-standard social and environmental accounting disclosures provided by companies. According to Zyznarska-Dworczak (2018), the legitimacy theory explains the behaviour of institutions in developing, implementing, and communicating corporate social responsibility programs and policies. This requires achieving the corporate social contract through the adoption of CSR that affects various activities, including assurance and sustainability activities.

In our sample, eight studies have applied this theory. Buallay and Al-Ajmi (2020) discussed the extent to which the features enjoyed by the audit committees have an impact on the sustainability reports of banks in the Arab Gulf countries based on four theories, including the theory of legitimacy. The study results showed a non-positive correlation between the financial experience of members of the audit committees and sustainability reports. Moreover, it indicated the importance of the positive role of the independence of audit committees members and the frequency of their meetings in determining the level of disclosure. The results also revealed the positive impact of the quality of auditors, and the size and the age of the bank on sustainability reports. Other seven studies have applied this theory (Boiral et al. 2019; Fernandez-feijoo et al. 2017; Hermawan & Gunardi 2019; Suttipun & Saelee 2015; Qingliang Tang 2019; Velte 2018).

One of the limitations of the theory of legitimacy is its consistency and vision that the processes associated with reporting and confirmation are formed through the pursuit of social legitimacy, while the processes related to the principle of achieving transparency and the application of the issue with stakeholders are neglected (Boiral et al. 2017). Thus, the legitimacy theory may be limited in its ability to explain how assurance-related service providers can confer and explain legality in scientific terms compared to specific ethical issues and behaviour associated with their audit activities (Boiral et al. 2019).

##### Institutional theory

The use of institutional theory includes the benefit of social and environmental accounting research from the point of view of different approaches and lenses (Bebbington et al. 2008). The institutional theory provides stronger results and interpretations than the theory of legitimacy, as it considers all the practical and internal factors of the subject under discussion. Moreover, it helps researchers to benefit from the theoretical interpretation that includes abundant information (Adams and Larrinaga-González 2007). Four studies from the sample discussed in this study used the institution theory. Rika (2009) used institutional theory to discuss the motivations for using environmental assurance in public sector organisations. Silvola and Vinnari (2021) discussed and clarified the role auditors play in promoting and ensuring sustainability among society. Despite the relevance of this theory, several studies have demonstrated that it is ineffective when multiple operations exist due to the external and internal environments of multinational corporations and large companies (Krajnovic 2018).

#### Methods applied in prior studies

The research methods that were followed in the previous studies were divided into five methods, including questionnaires and other empirical, annual reports, interviews and case studies, review and non-empirical research, and mixed-method studies. As shown in Table 4 (B), the review and non-empirical research is widely applied in the literature with 32 articles. These studies have discussed the sustainability assurance reports in public sectors (Handoko et al. 2020), corporate social responsibility assurance (Morimoto et al. 2004), application of environmental assurance (Westlake & Diamantis 1998), and internal audit and sustainability (Victoria Stanciu 2014). According to previous studies, the demand for sustainability report audits enhances the image of an institution and adds value to organisations’ planning, structure, monitoring, and accountability.

In our sample, 32 studies used archival data indicating that there is a great interest in conducting research based on realistic data from the reports of institutions or companies listed under the stock exchange markets. Ghani et al. (2018) reported that the samples that are used from firms reports have strong and accurate characteristics. Although many constructs (philosophy, culture, and preference of auditor) cannot be easily observed from an archival database, a significant number of practical works relied severely on archival data pointing to the need for more reliable and valid hand-collected research. Similarly, qualitative research has also received less attention from scholars where seven studies only have applied this method; out of these six articles, five studies have utilised interviews (Annuar & Abdul Rashid 2015; Boiral et al. 2020; Rennings et al. 2006; Silvola & Vinnari 2021), while the other two studies were case study research (Coetzee et al. 2019; Watson & Emery 2004). The investigation highlighted the lack of studies based on primary data (interviews and questionnaires) compared to secondary data. Future studies may use this method as one of the most important ways to obtain data in the social philosophy, culture, preference, and economic aspects (Roopa & Rani 2012).

#### Organisations focus

Concerning the unit of analysis, the literature was classified based on the sector under investigation as it might be useful in highlighting the parties interested in achieving effective sustainability assurance. This classification was applied in several previous studies (e.g. Guthrie et al. 2012; Kotb et al. 2020) as it helps to identify the institutions in which the assurance and sustainability research was conducted. Table 4 (C) shows that the (general/other) classification is obtained in most prior research with 54 articles where the study did not specify the type of institution that was investigated. Moreover, other studies focused on the public sector institutions (18 studies) and listed firms (13 studies). Some factors may help in obtaining data from the public sector due to the desire of the government sector to encourage researchers and accessibility to the information of listed corporations. Surprisingly, one study discussed the role of assurance in promoting sustainability in the private sector and this warrants future investigation. Furthermore, future studies may examine the role of assurance in promoting sustainability, relying on data from non-profit companies.

#### Sustainability definitions

The extensive and various literature on sustainability suffers from missing common agreement on accurate definition of sustainability as highlighted by several scholars (i.e. Chancé et al. 2018; Elkington 1998; Kuhlman & Farrington 2010; Munier 2005). Sustainability consists of three dimensions: the environmental dimension, the social dimension, and the economic dimension. Likewise, Ackers (2011) stated that sustainability assurance depends on three principles: the social principle, the environmental principle, and the economic principle, which require measuring them using specific indicators or criteria in accordance with the particular sustainability guidelines. Yet, some studies have proven that sustainability can be defined through two dimensions: the environmental dimension and the welfare dimension. Others, Kuhlman and Farrington (2010), argued that the separation between the economic and social dimensions is not logical because they are one concept that aims to achieve the welfare of the community. Thus, we categorised the research as follows; (i) environmental, social, and economic, (ii) environment and economic, (iii) environmental and social, and (v) environmental.

Results in Table 4 (D) show that the most comprehensive definition of sustainability received the largest number of research, which reached 52 papers (e.g. Guidara et al. 2021; Paterson et al. 2019; Slobodyanik & Chyzhevska 2019), followed by environment and economic with 23 studies. Moreover, some studies discussed sustainability from an environmental and social aspect with 16 studies, while three studies focused on the environmental aspects only. For example, Tang (2019) focused on the Chinese carbon audit institutions and showed that the most important factors that increased the practice of carbon assurance in China are the rapid expansion and development of carbon enterprises and the government’s encouragement of these enterprises.

### Thematic and content analysis of sustainability assurance research

The investigation showed that previous studies had examined a wide range of aspects related to sustainability assurance. These studies have focused on internal audits (7 articles) such as the role of internal audit functions (Desimone et al. 2020; Soh and Martinov-Bennie 2015), perceptions of internal auditors towards sustainability development (Desimone et al. 2020; Gray et al. 2014; Shih et al. 2006), external audit which discussed in one article (Ahmed 2016b), ACs (13 articles) such as characteristics of ACs (Al-Shaer and Zaman 2018; Buallay and Al-Ajmi 2020; Zaman et al. 2021), the experience of the member of ACs (Velte 2018), environmental assurance (10 articles) such as (He et al. 2015; Rennings et al. 2006; Nacanieli Rika 2009), and other specialist assurance (14 articles) such as financial audit (Canning et al. 2019), social audit (Gao and Zhang 2004; Zenad and Hasaballah 2020), carbon audit (Csutora and Harangozo 2017; Y. Zhang et al. 2019), government audit (Slobodyanik and Chyzhevska 2019), and audit in general (54 articles). However, these themes have been less examined in the literature, and more work addressing them is needed.

#### Internal and external audit and sustainability

The investigation indicates that seven articles have examined the relationship between internal audit functions and their role in achieving sustainability, while only one study investigated the role of external audits and their role in promoting sustainability. Table 5 summarises the objectives and results of studies in this area. The literature primarily from the accounting field has reported that the internal audit functions have worked to enhance and ensure sustainability and to provide some services such as assurance and consulting, which is consistent with international sustainability programs (Ridley et al. 2011). Internal audit functions help achieve sustainability by investigating data, investigating the validity and consistency of reports, and building trust with management, investors, employees, and stakeholders (Anagement et al. 2015). This is in line with the guidance provided by the Institute of Internal Auditors to enable internal auditors to ensure and facilitate consulting services for all aspects of sustainability (Ridley et al. 2011). Soh and Martinov-Bennie (2018) reported that management support and external reporting of sustainability information are key factors associated with internal audit’s involvement in sustainability assurance and consulting activities.

**Table 5:** Sample of studies on the internal and external and sustainability

Author/s	Country/method	Research focus	Summary of the findings
Desimone et al. (2020)	Cross country questionnaire CAEs ML	Investigating the role of internal audit in assessing risks associated with sustainability reports	The results show that the internal audit functions are linked in all ways to achieve sustainability and assess the risks associated with it
Stanciu (2014)	Romania Review	Investigating the effect of the sustainable development of organisations on the work of auditors	The study found that there is little impact as a result of the Romanian companies' lack of interest in sustainability activities
Soh and Martinov-Bennie (2015)	Australia 100 questionnaires (84 CAEs, 16 IAS) Descriptive	Investigating the extent to which internal audit functions participate in achieving and ensuring environmental, social and governance activities	The results show that governance comes to the fore in terms of the participation of internal audit functions in achieving it, and then environmental and social issues
Soh and Martinov-Bennie (2018)	Australia 100 questionnaires CAEs ML	The influence of the characteristics of internal auditing and the practice of sustainability on the role that internal audit plays in consulting and achieving environmental and social security	The results show that one of the most important factors that the internal audit functions contribute to is the reporting of sustainability information
Ridley et al. (2011)	Non-empirical viewpoint	Examining the role of internal audit in achieving sustainability	The results show that the internal audit is still far from playing this role as a result of not being asked to do so
Gray et al. (2014)	USA and Canada 372 questionnaires Descriptive and covariance	Investigating the contributions of internal auditors in achieving sustainability activities	The study found that auditors' perceptions of their role are linked to the achievement of traditional activities away from providing advisory services
Shih et al. (2006)	Taiwan 109 questionnaires *U*-test and *T*-test	Investigating auditors’ differing perceptions of environmental assurance	The results show that auditors' perceptions of environmental assurance are similar
Ahmed (2016a, 2016b)	Saudi Arabia 113 questionnaires *F*-test, ANOVA, *T*-test	Investigating the perceptions of the internal auditors towards their actions in achieving sustainability	The study found that auditors believe that there are responsibilities that they should fulfil in order to achieve sustainability

*ML*, multiple regression; *CAEs*, chief audit executives; *IAS*, internal audit service

However, dealing with environmental management to guarantee effective CSR practices is still the most vital challenge that faces internal auditors (Deloitte 2018), as they need to support the management by providing the necessary recommendations to improve activities and implement the plans set by the institutions. Hence, organisations should promote and practice rigorous assurance by experts who have sufficient experience to carry out their work (Victoria Stanciu 2014). The sample literature revealed that the presence of risk assessment by internal auditors, industry, and internal audit function age are important factors in the sustainability audits involvement (Desimone et al. 2020). However, auditors believe that they should be more involved in green information technology activities, as their current involvement is limited to the traditional role as assurance provider, not as facilitators or consultants (Gray et al. 2014). Future studies may discuss the role of internal audit and external assurance in ensuring sustainability from several aspects, including the characteristics of internal, external assurance members, their independence, financial and accounting expertise, and salaries, as well as their relationship with the top management team and AC. For example, it has been highlighted that the interrelationships between internal auditors, external auditors, the board of directors, and audit committees have a crucial role to play in sustainability assurance (Buallay & Al-Ajmi 2020). Furthermore, it has been found that the transparency of the adjustment to auditing policy as a response to COVID-19 is incomplete at best (Auld & Renckens 2021). The question that remained unanswered is how will economic conditions impact private sustainability assurance post-COVID-19 crisis?

#### Audit committees (ACs) and sustainability

The investigation suggested that a major research area has been the ACs as part of corporate governance with 13 studies. Table 6 presents sample studies on the role and effectiveness of the characteristics of members of ACs in ensuring sustainability. The literature highlighted that the characteristics of the ACs such as independence and the size of the committee, in addition to the financial and accounting experience, are closely related to ensuring sustainability. It can be said that the ACs contribute to improving sustainability reports through the extent to which members of the committees enjoy the required qualities (Bravo & Reguera-Alvarado 2019). However, the results of a study by Buallay and Al-Ajmi (2020) concluded that some characteristics of ACs are negatively related to achieving sustainability such as financial experiences of the members of the ACs which negatively correlated with the sustainability practices and disclosure, while the independence of members of the ACs, and frequent meetings are positively associated with ensuring sustainability. Also, the size of the committee is found to have an inverse impact on sustainability disclosure (Adegboye et al. 2020). The independence of AC members and gender diversity among auditors is positively related to ensuring sustainability, while there is a negative correlation with the size of ACs (Adegboye et al. 2020). The research was taken a step further by Pucheta-Martínez et al. (2021), who evaluated the moderating role of gender diversity on the impact of ACs, which appeared to be statically significant. Due to the inconclusive findings of the prior studies on the role of AC attributes in sustainability assurance, future investigation is highly warranted in this area. Table 6 summarises the objectives and results of some studies in this area.

**Table 6:** Sample of studies on the audit committee and sustainability

Author/s	Country/method	Research focus	Summary of the findings
Zaman et al. (2021)	Australia and New Zealand 100 firms ML	Determinants and factors of ensuring sustainability	The results show that the characteristics of ACs contribute effectively to ensuring sustainability
Buallay and Al-Ajmi (2019)	GCC 295 observations from 59 banks ML	Investigate the extent to which sustainability reports are affected by the characteristics of members of ACs	Independence and frequent meetings play a significant role in improving and enhancing the sustainability reports, while there is no impact on the ACs members’ experiences on the sustainability reports
Bravo and Reguera-Alvarado (2019)	Spain 375 observation reports ML	Determine the impact of women’s representation in ACs on ensuring environmental and social activities	The results show that diversity among ACs members in terms of gender has a positive impact on environmental and social reports
Cancela et al. (2020)	Spain 99 firms GMM	The study aims to determine the extent to which sustainability is affected by the characteristics of corporate governance	The study showed that the presence of the ACs is one of the most common factors that contribute to enhance sustainability
Al-Shaer and Zaman (2018)	The UK 333firms ML	Defining the role of ACs in ensuring sustainability	The study found that the characteristics of ACs play a key role in ensuring sustainability, especially with regard to independence
Velte (2018)	The EU 215 firms ML	Determining the impact of ACs members' experience on preparing sustainability reports appropriately	The study found that the impact of the financial and accounting experiences of members of the ACs has a strong positive correlation with the quality of sustainability reports
Hamidah and Arisukma (2020)	Indonesia 106 observations from 35 firms ML	Determining the impact of ACs as part of corporate governance on the level of sustainability	The study concluded that the size of the ACs mediates a positive relationship between the level of sustainability and corporate governance, while the result showed the absence of this effect on the independence characteristic
Annuar and Abdul Rashid (2015)	Malaysia 27 interviews Descriptive	Investigate the extent to which non-independent executives follow the laws and regulations governing their work	The results reveal that if the ACs follow the regulations governing their work, they will contribute to preserving the interests of investors in a way that promotes the development

*ML*, multiple regression; *GMM*, generalised method of moments

#### Other types of assurance and sustainability

Table 7 shows the studies that discussed other types of sustainability assurance such as carbon disclosure assurance (Kumar et al. 2021; Qingliang Tang 2019), shariah audit (Sulaiman & Alhaji Zakari 2019), social assurance (Gao & Zhang 2004), environmental assurance (Watson and Emery 2003), and auditing firm (Bostan et al. 2021; Coetzee et al. 2019; Ghani et al. 2018).

**Table 7:** Sample of studies on other type of assurance and sustainability

Author/s	Country/method	Research focus	Summary of the findings
Tang (2019)	China Critical Carbon auditing	Identify the factors that contributed to the increase in the demand for carbon audits	The results show that the increasing establishment of carbon organisations and the encouragement of state financing were the most important reasons for the increase in the demand for carbon audits
Sulaiman and Alhaji Zakari (2019)	Malaysia 7 firms Shariah audit	The study aims to measure financial sustainability from the perspective of Sharia audit	The study concluded that one firm out of the entire sample enhanced sustainability
Canning et al. (2019)	Netherlands-Belgium Case study and interviews Financial audit	How can financial auditing be practiced in non-financial institutions and its role in promoting and ensuring sustainability	The results show that assertiveness flexibility is able to address the relative importance of ambiguous data related to sustainability
Gao and Zhang (2006)	Critical/theory Social auditing	Determining the extent of the possibility of applying social auditing in order to achieve the sustainability sought by stakeholders	The study concluded that there is a positive correlation between liability audit and sustainability in terms of striving to enhance environmental, social and economic performance
Watson and Emery (2003)	UK Content analysis Environmental auditing	The study aims to assess the environmental audit in the UK	Analysis shows that the UK does not have an environmental code
Rika (2009)	Fijis Case study and interviews Environmental auditing	Aims to know the motives and reasons for the use of environmental auditing in Fijis	The results show that external and internal factors necessitated the use of environmental auditing, including the request of international organisations and the United Nations, in addition to the new laws in the country
Coetzee et al. (2019)	3 case studies Auditing Firms	The study aims to discuss how medium and small auditing companies practice their business in order to ensure the achievement of sustainability under different environmental conditions	The results show that the practice of small and medium-sized corporations to operate in accordance with regulations enhances their role in sustainability
Ghani et al. (2018)	Malaysia 222 reports for 74 firms Auditing firms Regression	Determine the factor affecting the sustainability	The study found that one of the factors is the size of the audit firms

##### Social assurance

The concept of social assurance is one of the types of audits that are concerned with monitoring, evaluating, and measuring general social (Carroll & Beiler 1975). Social assurance is defined as a set of organisational procedures that undertake the tasks of evaluating the social performance according to the expectations of firms and stakeholders (Elkington 1997). In general, social assurance can also be viewed as a function of examining the dynamic processes that organisations follow to enhance and improve the social performance from planning, inclusion, reporting, and stakeholder involvement (Zhang et al. 2003). According to Gao and Zhang (2006), the role of social accounting in ensuring and promoting sustainability lies in the extent of consolidation and complexity of relationships with stakeholders, which enables the establishment of the competitive advantage of the company based on sustainability. Several social issues involved in the assurance and/or consulting activities of corporations include customer privacy, product responsibility, donations and sponsorships, community impacts and relations, human rights, supply chain issues, training and education, employee retention and turnover, and occupational health and safety. Researchers argued that governance and environmental issues are the greatest current and future importance of assurance activities, while social issues appeared to be overlooked (Soh & Martinov-Bennie 2015).

##### Environmental assurance

It could be argued that environmental assurance is one of the voluntary activities of companies (Radu et al. 2012). According to Dhariwal (2013), environmental assurance is one of the management tools that enhance the overall environmental performance of corporations. A study by Khodjaeva (2019) demonstrated the importance of environmental assurance as a tool that contributes to increasing and improving investment attraction and working to reduce the destructive resources of the economy.

Similarly, Watson and Emery (2003) suggested that environmental assurance is a set of sub-reports (corporate responsibility reports) that express many activities of organisations. With regard to environmental assurance, it has been found that there is a strong correlation between environmental assurance and CSR (Watson and Emery 2003). Thus, environmental assurance may contribute to the achievement of sustainable development goals when viewed through the lens of a policy orientation that emphasises the importance of preserving the environment while balancing economic development needs. Thereby, it contributes to the development and planning processes’ non-negative impact on society via the environment. Additional research is needed in this area.

##### Carbon assurance

Carbon assurance is one of the new jobs that have been practiced in many developed countries such as China ( Zhang et al. 2019). Chen and Mei (2012) pointed out that carbon assurance is one of the branches of environmental assurance and it shows how countries can adapt to shifts in economic development, which contributes to strengthening and improving national auditing. Similary, Tang (2019) referred to carbon assurance as an extension of the comprehensive and general idea of sustainability or environmental assurance. Likewise, his study showed that there is an increase in the application of carbon auditing due to the economic development in some countries. This necessitated the application of this carbon auditing to work towards achieving a balance between growth in domestic products and the protection of the ecosystem, considering that carbon auditing is a tool through which innovation governance can be managed, transformations management and sustainable technical, social, and organisational transformation.

It should be noted that the carbon assurance differs from the traditional audit assurance, which depends on processes that cover revenues and expenses, review of laws, financial expenditures, internal management and reports, while carbon audit covers the audit of carbon derivatives and what is related to it. In the sample that discussed the carbon audit, studies were limited to China and India. Therefore, the economic development and industrial transformations in these two countries are among the most important factors that necessitated the application of this type of guarantee. Interestingly, there are no studies on the application of carbon insurance in countries such as the USA and Eastern countries of Asia, which are also experiencing industrial transformations, which may require the application of this type of assurance.

##### Sharia assurance

Sharia assurance is one of the most important aspects practiced in Islamic institutions including Waqf institutions. It is the most important way to measure the level of commitment and compliance with Islamic law principles. According to Khalil et al. (2014), the Islamic Waqf institutions contributed positively to achieving economic and social development in some Islamic countries such as Egypt, Kuwait, and Malaysia. Furthermore, only one study observed that the practice of Sharia assurance contributes to adopting advanced governance and Sharia assurance mechanisms, which contributes to facilitating and promoting sustainable development and economic growth (Mohammed et al. 2020). In Table 7, we presented the objectives, results, methods, and type of audits of some of these studies.

## Future research avenues

Several topics were identified for future research based on the SLR of sustainability assurance literature that may be explored via novel theoretical approaches or empirical methods. The investigation revealed that sustainability assurance is a recent topic and there has been growth in the number of published studies addressing this topic in the last few years. In terms of the geographical distribution of studies, the review showed that China is the most investigated market regarding the role and importance of assurance and the extent of the need for sustainability assurance in institutions. However, due to the lack of research on some markets, there is a need for more research investigating sustainability assurance in both developed and developing countries that received less attention in the literature. Guidara et al. (2021) called for further work on emerging economies with significant challenges facing auditing practices. It may be instructive to conduct in-depth investigative studies in European and Arab countries, in which available studies were few. It would also be interesting to conduct analytical studies on the Chinese and American cases, which have not been adequately studied according to the results of our study. Researchers could also discuss environmental assurance and carbon assurance, especially in countries that enjoy great economic development. Comparative research utilising data from different legal jurisdictions would be useful in helping the intellectual understanding of institutional and legal environment influence sustainability assurance (Al-Shaer & Zaman 2018).

Surprisingly, only one study has discussed the role of auditing in promoting sustainability in the private sector, which warrants future investigation. Also, future studies may discuss the role of auditing in promoting sustainability, relying on data from non-profit companies or waqf institutions (Sulaiman & Alhaji Zakari 2019).

Moreover, it has been found that empirical studies based on questionnaires and interview tools are very limited, pointing to the need for more research using these approaches. Future studies may use the survey method as one of the most important ways to obtain data in the social philosophy, culture, preference, and economic aspects (Ghani et al. 2018; Roopa & Rani 2012). Al-Shaer and Zaman (2018) argued that utilising interview techniques would provide in-depth insights into the role of auditing relating to sustainability in certain organisational settings. It is necessary that academics continue to employ suitable techniques to attenuate endogeneities issues, though we are inspired by the growing attention paid to methodological concerns of endogeneities.

From our SLR, another area with negligible prior research is the development of a theoretical framework (e.g. Ruiz-Barbadillo & Martínez-Ferrero 2020). Studies that are based on theories may provide results and insights with strong contributions to the understanding and development of the topic. To expand theoretical perspectives to explore the relationship between audit and sustainability and to show the important role of sustainability audit towards stakeholders, we encourage future research to consider applying theoretical grounds in further exploration of the sustainability audit topic. In light of the current conditions and as a result of the environmental changes that occurred during the COVID-19 crisis, studies can be expanded to discuss the importance of sustainability assurance in such crises.

Furthermore, our review showed that the absence of studies devoted to investigating the impact of internal and external auditing. It seems that there is an expansion in the studies that discussed the characteristics of members of the ACs on sustainability. Future studies may discuss the interactive role of internal and external audits in ensuring sustainability (Al-Shaer & Zaman 2018), in addition to the possibility of discussing the characteristics of audit members. For example, Wang et al. (2020) highlighted the need for more work on the role of auditor gender preferences toward sustainability practices. Also, it has been reported that the growing call for assurance and the expanded risk of auditor’s litigation during the COVID-19 epidemic would increase the’s effort and working time of auditors. How will the growth in demand influence sustainability auditing post-COVID-19 crisis in terms of fees and assurance quality?

On the other hand, and in light of the great need to monitor sustainability activities, assurance activities may be one of the basic tools for this, which requires the development of assurance standards in line with the needs of stakeholders in achieving sustainability. Few studies discussed this topic, perhaps the most recent of which was published in a *Sustainability Journal* by Fraser et al. (2020). The question that wants to be answered is whether the common standards can achieve the stakeholders’ goals in achieving sustainability in light of the increasing need to implement sustainability activities and in light of changing environmental conditions such as a COVID-19 crisis or there is a need to issue other standards that go along with these environmental changes. Future studies may look into this area with a focus on the possibility of conducting investigations with regulators, audit and sustainability institutions, and stakeholders.

In considering context and process, researchers can unravel different dimensions of auditor attributes, such as auditor tenure (assurance providers), opinion, market competition of assurance, assurance qualification, audit rotation and unpack how they affect sustainability activities and disclosure (Chiang & Torng 2015; Ruiz-barbadillo & Martínez-ferrero 2020; Tahinakis & Samarinas 2017).

In addition, there is a need to better account for audit sustainability systems as a result of environmental changes that stop the practice of audit systems in their natural form such as the COVID-19 crisis which has affected all economic sectors (Castka et al. 2020; Johnsson et al. 2020; Khatib & Nour 2021). For instance, is resilience operational linked to changing audit methods and procedures, or should some solutions complement the work between traditional audit systems and new systems implemented in light of environmental changes in a way that ensures the achievement of sustainability? Hence, this is another area that needs more studies to consider for the impact of auditor resilience on audit quality.

## Conclusions and recommendations

Sustainability is a rapidly growing topic among firms, society, and academics. Nonetheless, there is a dearth of empirical and review studies discussing the role of assurance in achieving and assuring the sustainability of institutions. This study, therefore, followed a systematic literature review to provide a comprehensive view of the role of audit in achieving sustainability by using a final sample of 94 studies.

The study revealed that the need for audits for sustainability reports does not only enhance the reputation of institutions but also adds more value to the organisation’s planning, structure, monitoring, and accountability. This paper adds to the existing literature on the audit and sustainability of corporations by offering a comprehensive review of the existing literature. It highlights the role of auditing in enhancing the practice of sustainability to the extent of the stakeholders’ desire and confirms that there is a significant role for auditing in emphasising sustainability as the most important means contributing to mitigating the risk related to environmental violations. In general, the results showed that the role of audit in promoting and ensuring sustainability is crucial, especially if the audit characteristics are different. The findings may help strengthen the understanding of parties such as regulators, practitioners, and potential investors on the intellectual development of the sustainability audit field and allow the development of new and remarkable empirics in future research.

The current sustainability assurance standards need to be revised to enhance the professionalism of assurance practices. As a result of different aspects/research subjects of sustainability assurance, auditors should clarify the criteria used and systematically refer to established standards that enhance the credibility of their verification and the readability of assurance statements, as without such standards would there be great variability in the wording used within the conclusions and assurance statements. Furthermore, the revision of assurance standards should involve stakeholders who are deeply concerned with improving the quality and reliability of sustainability assurance, irrespective of the commercial and procedural aspects of these standards.

Similar to other studies, this research is not without limitations. We utilised several keywords to identify the sample literature in the Scopus database as it is the wide abstract indexing source of peer-reviewed articles. Future research, however, could consider other databases such as Web of Science, ABS, and ABDC. Moreover, the search method applied in this study was restricted; therefore, the results of the search string used in this paper may not cover all documents in this area. Hence, similar studies in future could add other related keywords to the search string such as environmental performance or disclosure.

## Data Availability

Not applicable.
